# Living on the Edge: Social Exclusion and the Receipt of Informal Care in Older People

**DOI:** 10.1155/2016/6373101

**Published:** 2016-11-01

**Authors:** Lena Dahlberg, Kevin J. McKee

**Affiliations:** ^1^School of Education, Health and Social Studies, Dalarna University, Dalarna, Sweden; ^2^Aging Research Center, Karolinska Institutet & Stockholm University, Stockholm, Sweden

## Abstract

Older people have been identified as being at risk of social exclusion. However, despite the fact that care is commonly required in later life and the majority of that care is provided by informal carers, a connection between social exclusion and informal care-receipt has rarely been considered. The aim of this study was to examine how informal care-receipt is related to social exclusion. A face-to-face questionnaire survey on social exclusion and informal care-receipt was carried out among older people (*n* = 1255) living in Barnsley, United Kingdom. Multivariable analyses examined the association between social exclusion and categories of informal care-receipt: care-receiver; assurance-receiver; nonreceiver with no need; and nonreceiver with need. Compared to being a nonreceiver with no need, participants were more likely to be care-receivers or assurance-receivers if they had higher levels of social exclusion. The highest level of social exclusion, however, was found in nonreceivers with need. Despite a lack of informal care and support, formal practical support and personal care were also low in this latter group. Findings are discussed in relation to the conceptualisation of care-receipt and how contact with medical services could be an opportunity for identification and appropriate referral of nonreceivers with need.

## 1. Introduction

A policy agenda at the European level is the reduction of social exclusion [1–3], defined as a “process whereby certain individuals are pushed to the edge of society and prevented from participating fully” [4]. Social exclusion is associated with reduced quality of life and health deterioration [5, 6]. Older people have been identified as a population subgroup at particular risk of social exclusion (e.g., [7]). Every second older person in England is experiencing social exclusion [8] and in deprived urban areas in England two-thirds of older people experience social exclusion [9]. While social exclusion has been explored in relation to a number of factors, including income, health, and disability, it has rarely been considered in relation to informal care-receipt. This is despite the fact that with increasing age older people require greater amounts of care and support and that informal care comprises the majority of this care [10]. Furthermore, research has shown that the transition to requiring care can reinforce social exclusion (cf. [11]). This paper considers how dimensions of, as well as risk factors for, social exclusion are linked to informal care-receipt in older people.

Social exclusion is a multifaceted concept covering dimensions such as production activity, financial activities, social relations, social activity, and political/civic activity [12, 13]. Lack of participation in or access to activities/services specified by these dimensions can be seen as indicators of social exclusion. Within social exclusion research concerning older people Scharf and colleagues [6] propose that older people may experience social exclusion on the following dimensions: social relationships; civic activities; basic services; neighbourhoods; and material resources. By comparison a United Kingdom (UK) government report [8] defines social exclusion of older people in terms of exclusion on the following dimensions: social relationships; cultural and leisure activities; civic activities; basic services; neighbourhoods; financial products; and material goods. As can be seen, although there are different operationalisations there is consensus around core dimensions of social exclusion for older people. What is apparent is that in social exclusion research on older people there is less emphasis on production activity and more emphasis on the importance of neighbourhood, also often discussed in terms of community. Many older people have spent a substantial period of their lives in a particular neighbourhood, have strong emotional investments in the surrounding community, increasingly rely on neighbourhood relationships for support in old age, and also tend to spend more time than younger people in the immediate neighbourhood [14].

Research exploring risk factors for social exclusion among older people has identified an enhanced risk of social exclusion with increasing age and ethnic minority origin and, among those living alone, having no children, and being on low income [6, 8]. Poor health, long-standing illness, and depression have also been found to increase the risk of social exclusion ([6, 8, 15], cf. [16]). Negative consequences of poor health can to some extent be compensated for by access to social and health care [17], thereby helping to prevent social exclusion. A few studies have focused on access to formal care in relation to social exclusion [11, 18–20], but research on access to* informal* care (i.e., care provided by friends and family) in the context of social exclusion is very limited. This is surprising, since as noted previously the majority of all care for older people in the community is informal and recent research indicates that the level of informal care is increasing [21, 22].

As people age, a reduction in functional capacity occurs that increases their need for help with activities of everyday life [23]. While the level of care received by an older person might be expected to map onto their need for care (due to, e.g., functional limitation and frailty), there are many factors that can disrupt this mapping resulting in unmet need for care in a significant minority of older people [24–26]. Unmet need can have serious consequences for an older person including increased mortality risk [27] and also prevent them from participating fully in society. Thus, unmet need for care could arguably place an older person at risk of social exclusion.

The relationship between care-receipt and social exclusion could be hypothesised to take different forms. It could be argued that the level of care-receipt if directly mapping onto the level of need in the older person would be a marker for his/her level of social exclusion, given that higher levels of care received would be in response to higher levels of illness or frailty, that is, risk factors for social exclusion. Yet care-receipt is linked to indicators of social* inclusion* such as the availability of, as well as access to, social networks, so an alternative hypothesis is that higher levels of contact with family and friends as a result of being a care-receiver might serve to reduce social exclusion. There is also the critical issue of where the level of care-receipt is not appropriate to need: where need for care exists but care is not received, is the degree of social exclusion particularly high?

This paper aims to examine the relationship between social exclusion and the receipt of informal care and reports the analysis of relevant data from the Barnsley Social Exclusion in Old Age Study, which sought to explore social exclusion among older people via a survey of over 1,000 respondents (see also [28]).

## 2. Materials and Methods 

### 2.1. Design and Sampling

A questionnaire survey was carried out in the metropolitan area of Barnsley, England, UK. Barnsley was selected as the study site since it encompasses both urban and rural areas, allowing the exploration of how social exclusion processes might differ in such contrasting areas. To ensure adequate cell size at subgroup level for specific analyses, a sample of *n* = 600 for each of the two areas (i.e., *N* = 1200) was proposed, providing good statistical power for the analyses reported in this paper. Sampling occurred from seven electoral wards with an urban profile and from 16 electoral wards with a rural profile. Within each electoral ward households (which included supported accommodation) were randomly selected via local electoral registers. With oversampling of households required in order to obtain sufficient participants, a total of 11,035 households were sampled.

### 2.2. Participants

Potential participants were ineligible if they were under 65 years of age and were excluded from the study if their physical and/or mental health was too poor to allow them to complete an interview or respond to questions reliably; 59 individuals were excluded from the study as a result of this latter criterion. Only one older person was recruited per household regardless of whether more than one older person resided at a given address. In total 1,255 older people participated in the study, of whom 6.5% were recruited from supported accommodation. The response rate was 68.1% and did not differ significantly between urban and rural areas.

### 2.3. Materials

A questionnaire was developed that addressed a range of indicators of, as well as risk factors for, social exclusion, together with the topic of care-receipt. Given the potential frailty of some respondents, the need to keep the questionnaire concise meant that brevity was a key criterion during instrument selection. On occasion items and scales were adapted to more precisely address the study population or agenda.

An item used in the EUROFAMCARE study [29] to identify informal carers was adapted to produce a categorical variable of* informal care-receipt*: “Do you rely on a friend or relative (including your partner or other people in your household) to provide you with care or support for four hours per week or more?” (response categories:* yes*/*no*). Those participants responding “yes” were categorised as* care-receivers*. Participants who responded “no” were asked: “Do you have someone who looks in on you to see if ‘everything is all right'?” (cf. [30]) (the three response categories were* yes*;* no*,* no need*; and* no*,* despite need*). Those participants responding “yes” were categorised as* assurance-receivers*; those responding “no, no need” were categorised as* nonreceivers without need*; those responding “no, despite need” were categorised as* nonreceivers with need*. This procedure therefore produced a four-category variable of care-receipt.

In this article, the following dimensions of social exclusion were considered: financial resources; social relationships; community; and social engagement.

Financial resources were operationalised via the following item measuring income discomfort: “Which of these descriptions comes closest to how you feel about your household's income nowadays?” with response options ranging from* very comfortable on present income* (1) to* very difficult on present income* (5) [31].

Social relationships were measured via items on social contacts, informal caregiving, and loneliness. For data on social contacts a question asked “how often do you meet and spend time with any of the following people?” Independent responses were required for family members (six categories), neighbours, and friends [32, 33]. For family member contact responses were coded as* weekly contact or more *(1) or* less than weekly contact *(0) for each category and items summed to indicate overall level of contact (scores ranging from 0 to 6, high scores indicating high contact). For the two categories of nonfamily members responses were combined and coded for analysis as* no contact* (0),* at least twice weekly contact with friends or neighbours* (1), or* at least twice weekly contact with friends and neighbours* (2).

The informal care item in the EUROFAMCARE study mentioned above was used in its original form for measuring informal caregiving, that is, “Have you a friend or relative (including your partner or other people in your household) who relies on you to provide them with care or support for four hours per week or more?” (response categories:* yes*/*no*).

Loneliness was measured by the De Jong Gierveld Loneliness Scale, in which respondents indicate the extent to which 11 statements relating to loneliness apply to their situation and the way they feel now, with response options* yes*,* more or less*, and* no*. The items are scored in relation to two subscales: Emotional Loneliness (scale range 0–6, sample Cronbach *α* = .81) and Social Loneliness (scale range 0–5, sample Cronbach *α* = .76) [34].

Respondents were asked to what extent they agreed with each of 13 statements about their local community, defined as “within 20 minutes' walk or about a mile from home.” Example items are “I feel really part of this area”; “Vandalism and graffiti are a big problem in this area” (response scale from* strongly agree *(1) to* strongly disagree *(4) [7, 8, 35]).

For data on social engagement participants were asked, first, to consider for “how often, if at all, do you engage in the following activities?” and record a response for twenty different activities. Example activities are “Go out for a meal”; “Attend leisure activities (e.g., dancing, bingo or attend a social club)”; “Tend to the garden or allotment” (response scale* several times a week* (6) to* never* (0)) and, second, to indicate for each applicable activity if it is performed* usually with somebody* (3),* sometimes with somebody* (2), or* usually alone* (1) [32, 33]. The product of participants' engagement scores and socializing scores resulted in a single “social engagement” score for each activity.

This study considered the following risk factors for social exclusion: sociodemographic characteristics; health; and well-being. The questionnaire contained standard items addressing sociodemographic characteristics: age; gender; marital and coresident status; ethnicity; and duration of local residence. Education was measured by an item with six response categories, merged into two broader categories for analysis: “low education” (highest level,* completed school*,* no qualification/certificate*) and “medium to high education” (lowest level,* completed school with qualification/certificate*).

Self-reported health was assessed via the item: “In general, would you say your health is…” [31] measured on a five-point scale (*excellent *(1) to* very poor *(5)). Psychological well-being was measured using the World Health Organisation-5 Well-being Index [36] (WHO-5; scale range 0–25 (high score = high well-being); sample Cronbach *α* = .87).

Finally information on formal care-receipt was gathered via items asking if the participant had in the last month received medical care, personal care (e.g., from a district nurse), or practical support (e.g., from social services, home help, and warden); response categories for all items were* yes*/*no*.

### 2.4. Procedure

In order to ensure standardisation of data collection procedures and maximise interviewer sensitivity to reliability issues (e.g., physical or mental health problems in respondents, the influence of people present during interviews), interviewers were provided with training commensurate with their prior experience. Training therefore amounted to a few hours (for experienced interviewers) to two days (for inexperienced interviewers).

Upon selection, a household was sent a letter presenting the purpose of the study. An interviewer subsequently visited the address to establish whether anybody in the household was 65 years or older and, if so, whether this person was willing to participate in an interview. Each interview lasted on average 50 minutes. Seventy-eight percent of the interviews were conducted alone with the interviewee, while the rest were carried out with the interviewee accompanied, usually by a family member.

Interviewers completed several items at the end of the questionnaire as a quality check addressing whether or not the respondent had tried to answer the questions to the best of his or her ability; whether the respondent understood the questions; and whether anyone was present during the interview that could have interfered with the interview. There was also an option for the interviewer to write additional information about the interview. Where data drawn from these items indicated a problem with the interview, researchers discussed the problem with the interviewer, and if there was a suggestion that the reliability of the data could be suspected, the interview was excluded from the study.

### 2.5. Data Analysis

Data were analysed using the IBM Statistical Package for Social Science (SPSS) 22.0 for Windows.

Scale development occurred for items on perception of the local community and social engagement, utilizing principle components analysis and reliability (Cronbach *α*) analyses with item trial removal. Three subscales of perceptions of the local community were developed: Perceived Community Trust (3 items, M = 11.4, SD = 1.89, and *α* = .68); Perceived Community Integration (4 items, M = 15.6, SD = 2.39, and *α* = .69); and Perceived Community Security (3 items, M = 9.53, SD = 3.27, and *α* = .80). Two reliable subscales of social engagement were developed: Social, Cultural and Leisure Activity (7 items, M = 26.14, SD = 18.42, and *α* = .65) and Sport and Outdoor Activity (5 items, M = 12.40, SD = 13.06, and *α* = 0.56).

Bivariate analyses identified significant associations between the dependent variable (DV; the categorical care-receipt variable) and independent variables (IVs; indicators of and risk factors for social exclusion and other assessed variables). One-way ANOVA was performed for continuous IVs, with Hochberg's GT2 and Games-Howell tests as appropriate for post hoc analysis of group differences; Chi-square procedures were used for categorical IVs. A multinomial logistic regression was then performed to determine those IVs that predicted membership of the categories of care-receipt in a multivariable model. No adjustment to experimental alpha was made for multiple testing; significance for each test was set at *p* < .05. Given also the substantial sample size significant tests should be regarded cautiously and with thought to effect size.

## 3. Results

Care-receipt status and sample characteristics are summarised in Table 1. Regarding care-receipt 21.8 percent of the respondents were care-receivers and 24.1 percent were assurance-receivers, while 5.5 percent were nonreceivers with need and 48.6 percent were nonreceivers without need.

### 3.1. Bivariate Analysis

A brief summary of the bivariate analyses is described below, with a full presentation of the results of the analyses in Table 2. For the sake of concision IVs with nonsignificant associations with the DV are not presented.

At the top of the table bivariate associations between care-receipt status and social exclusion dimensions are presented. In post hoc tests nonreceivers without need had significantly lower dissatisfaction with household income than care-receivers and nonreceivers with need. Post hoc tests also showed that family contact was significantly lower, and Social Loneliness and Emotional Loneliness was significantly higher in nonreceivers with need compared to the three care-receipt categories, and the lowest proportion of older people with contact with friends and neighbours was also found in this group. Post hoc tests also indicated that nonreceivers without need were significantly lower on both Social and Emotional Loneliness than care-receivers and lower on Emotional Loneliness than assurance-receivers.

Other post hoc tests indicated that nonreceivers with need scored significantly lower on Perceived Community Integration than older people in the other three care-receipt categories. For Perceived Community Trust and Perceived Community Security post hoc tests indicated significantly lower scores for nonreceivers with need and care-receivers compared to assurance-receivers and nonreceivers without need. Finally, post hoc tests indicated that nonreceivers without need had significantly higher scores on Social, Cultural and Leisure Activity and Sport and Outdoor Activity than older people in the other three care-receipt categories.

In the next part of Table 2 analyses of risk factors of social exclusion are presented. Of the categorical IVs gender, coresident status, education, and place of residence were all significantly associated with care-receipt. Of the continuous IVs, in post hoc tests, nonreceivers without need were significantly younger than those in the other three categories. Post hoc tests determined that duration of local residence was significantly greater in care-receivers and nonreceivers with need than among assurance-receivers and nonreceivers without need. For measures of self-rated health and well-being post hoc tests indicated that nonreceivers without need differed significantly from those in the other three categories, having better self-reported health and better well-being. In addition, post hoc tests indicated that care-receivers had poorer self-reported health and well-being than assurance-receivers.

At the bottom of Table 2 associations between care-receipt and formal care use are presented. The highest level of medical care receipt was found among nonreceivers with need, with the lowest level in nonreceivers without need. This pattern differed in comparison with formal personal care-receipt and formal practical support-receipt as care-receivers and assurance-receivers were those with the highest proportion in receipt of these formal services.

### 3.2. Multivariable Analysis

The* nonreceivers without need *category was used as the reference category for the DV in the multinomial logistic regression. IVs entered into the regression consisted of those established through bivariate analysis to be significantly associated with care-receipt. The IV Social, Cultural and Leisure Activity was recoded from a 5-point to a 3-point scale to reduce the number of cells in the model. Following trial runs best model fit statistics were obtained when nonfamily contact and Perceived Community Security were deleted. Due to missing data model *n* = 980 (see Table 3).

In comparison to a constant-only model the model was reliable (*χ* ^2^(51) = 488.68, *p* < .001), with good model fit (model *χ* ^2^(2886) = 2905.15  *p* > .05, Nagelkerke *R* ^2^ = .43). Table 3 presents for all variables in the model the Wald test for significance of each coefficient and the odds ratio with 95% confidence intervals for each of the three comparisons between the three care-receipt categories and the reference category.

We consider first the prediction of membership in the* care-receiver *category relative to being in the reference category. Starting with social exclusion variables, a unit increase in Perceived Community Trust and Social, Cultural and Leisure Activity corresponded to 0.87 and 0.98 odds of being a care-receiver, and participants at the lowest and middle levels of Sport and Outdoor Activity had, respectively, 4.79 and 1.93 odds of being a care-receiver compared with participants at the highest level. Regarding risk factors of social exclusion, a unit increase in age corresponded to a 1.06 increase in the likelihood of being a care-receiver; participants in the rural group had 0.38 odds of being a care-receiver compared to the urban group; a unit increase in (poor) self-reported health corresponded to a 2.54 increase in the likelihood of being a care-receiver. Regarding formal care use, participants who had received medical care had 2.81 odds of being a care-receiver compared to those who had not received medical care.

Next we consider prediction of membership in the* assurance-receiver* category relative to the reference category. Regarding social exclusion variables, one-unit increase in family contact and Emotional Loneliness corresponded, respectively, to 1.21 and 1.16 increases in the likelihood of being assurance-receivers, while participants at the lowest and middle levels of Sport and Outdoor Activity had, respectively, 1.85 and 1.71 odds of being assurance-receivers compared with participants at the highest level. Regarding social exclusion risk factors, a unit increase in age corresponded to a 1.05 increase in the likelihood of being an assurance-receiver; male participants had 0.63 odds of being assurance-receivers compared to female participants; participants in the rural group had 0.49 odds of being assurance-receivers compared to the urban group; a unit increase in duration of local residence corresponded to 0.99 odds of being assurance-receivers; a unit increase in (poor) self-rated health corresponded to a 1.30 increase in the likelihood of being assurance-receivers. Finally, participants who had received medical care had 1.51 odds of being assurance-receivers compared to those who had not received medical care.

Lastly, we considered prediction of membership in the* nonreceivers with need *category relative to being in the category nonreceiver without need. Regarding social exclusion dimensions, analyses show that a unit increase in Social Loneliness corresponded to a 1.39 increase in the likelihood of being nonreceivers with need. Regarding risk factors of social exclusion, a unit increase in age corresponded to a 1.07 increase in the likelihood of being nonreceivers with need; participants in the rural group had 0.26 odds of being nonreceivers with need compared to the urban group. Finally, participants who had received medical care had 5.82 odds of being nonreceivers with need compared to those who had not received medical care.

## 4. Discussion

Our multivariable analysis produced a significant model that predicted older people's membership of care-receipt categories on the basis of social exclusion dimensions and a range of risk factors for social exclusion. The pattern of associations in the model had face validity. Greater age, recent contact with medical care, and poorer health, all being meaningful indicators of frailty and/or need for support in an older person, increased participants' likelihood of being an assurance-receiver and were associated with an even greater likelihood of being a care-receiver, relative to being a nonreceiver with no need. Greater age and poorer health are established risk factors for social exclusion [8]. Another variable significant in the model, Sport and Outdoor Activity, is included in the social exclusion dimension social engagement. The picture that emerges from the model is that older people with higher scores on dimensions of and risk factors for social exclusion were significantly more likely to be in receipt of assurance and care. Membership of the nonreceivers with need category was also predicted by indicators of frailty (greater age, recent contact with medical services) and an indicator of social exclusion (greater Social Loneliness). If one examines the pattern of bivariate associations between the IVs and the care-receipt variable, nonreceivers with need in comparison to older people in the other categories demonstrated the highest levels of Social and Emotional Loneliness and the lowest levels of income comfort, social contact (with family members, friends and neighbours), social engagement (Social, Cultural and Leisure Engagement), Perceived Community Integration, Trust, and Security, indicating that these older people experienced the highest levels of social exclusion. If the categories of care-receipt are considered as a continuum with nonreceivers without need as one end of the continuum, then nonreceivers with need would exist at the other end: the odds ratios for greater age and recent contact with medical services are larger for this category than for care-receivers and assurance-receivers.

However, other variables uniquely contributed to membership of different care-receipt categories, suggesting that nonreceivers with need, care-receivers, and assurance-receivers might differ as social positions or roles not just in terms of degree. A continuum model of care-receipt might therefore be insufficient. Lower Perceived Community Trust and Social, Cultural and Leisure Activity increased the likelihood that a participant was a care-receiver; being female, greater family contact, and greater Emotional Loneliness increased the likelihood that a participant was an assurance-receiver; and greater Social Loneliness increased the likelihood that a participant was a nonreceiver with need. When seeking to conceptualise care-receipt in older people, therefore, one model would be that the experience of care-receipt is a progression through increasing levels of support and care until in many cases the informal care network breaks down. A different model would be that whether one is a care-receiver or an assurance-receiver or a nonreceiver with need is not about the current occupation of one role within a progression of care-receipt, but rather an outcome of that role being more likely when other factors are true, for example, as where being female and having recent contact with one's family increase the odds that one is an assurance-receiver. Our findings suggest that both models coexist in later life.

### 4.1. Social Exclusion and Care-Receipt

An Irish study of care-receipt among older people [37] reported that 49% of older people received care over a 12-month period. The study used a broad definition of care-receipt, including people receiving care once weekly or less. This corresponds in our study to those participants in the care-receipt and assurance-receipt categories combined, which comprised 46% of our sample, a similar figure. The present study's focus on the concept of assurance-receipt is relatively unique: most studies explore the issue of caregiving among burdened carers, that is, those providing many hours of care each week, with few focusing on “light” carers, and by extension older people in receipt of only a few hours of care (cf. [38]).

A question posed in the introduction to this paper was whether being a care-receiver might be associated with lower levels of social exclusion, as care-receipt is almost by definition linked to contact with friends and family, an indicator of social* inclusion*. Our findings offered little evidence to support this conjecture. Being a care-receiver was not significantly associated in the multivariable model with greater contact with friends or greater contact with family. Furthermore care-receipt was predicted by lower Perceived Community Trust, suggesting that care-receipt might be linked to a poorer relationship with one's neighbourhood, another indicator of social exclusion. While one predictor of being an assurance-receiver was greater contact with one's family, this indicator of inclusion has a counterbalance in the association between being an assurance-receiver and greater Emotional Loneliness. Similar results have been found in research on formal care-receipt. Barrett et al. [11] argue that home-based formal care contributes to a disconnectedness of the care-receiver from self, family, home, and the broader community, thereby contributing to social exclusion (see also [20]).

In the multivariable analysis one of the key predictors of membership in the different care-receipt categories was place of residence with care-receipt being more common in urban areas. There is a lack of research on rural/urban patterns of informal care-receipt, and the findings from the existing research are contradictory. For example, in line with our findings, it has been suggested that American urban older residents are more likely to have informal support from kin and to have children living nearby, as there is a general pattern of outmigration of children from rural areas and an in-migration of older people to rural areas [39]. On the other hand, Canadian research indicates that informal care-receipt is more common among rural residents [40]. With regard to our findings, since several demographic, health, and social variables were controlled for in our analysis, we suggest it is likely a complex interaction of personal, demographic, and social factors related to residence in urban environments that produced high levels of care-receipt and an unmet need for assurance, relative to residence in rural areas. More research is needed in order to obtain a deeper understanding of the mechanisms behind rural/urban care-receipt patterns.

### 4.2. Policy and Practice Implications

One Spanish study on older people in need of help with activities of daily living identified a group of older people (5.9% among women, 7.9% among men) who did not receive help [26], and a Canadian study [25] found that approximately 2% of older people outside institutions experienced unmet care needs. In our study such a group was represented by the nonreceivers with need, which comprised 5.5% of our sample. This group was found to score highly on several dimensions of and risk factors for social exclusion (cf. [9]). Of interest is the fact that we found that older people with higher levels of care-receipt received more formal personal care and formal practical support. The exception to the pattern was the nonreceivers with need, where despite a lack of informal care and assurance there were also low levels of formal personal care and formal practical support. However, this group had the highest level of recent contact with medical services, a relationship also found in the Spanish study mentioned above [26]. Similarly, recent research has found that lonely older people have a higher use of health care services than nonlonely people [41] and that loneliness and social isolation are a common nonmedical problem presented by noncritically ill older people in emergency departments ([42], for a review, see [43]). One interpretation of this finding is that, in the absence of informal care and in the presence of need, older people will turn to medical services (perhaps primary care practitioners) in order to address their unmet need. Whatever the contributory factors, an opportunity exists during contact with medical care providers whereby identification and assessment could offer a route into referral for appropriate formal personal care and/or formal practical support. This recommendation is in line with previous calls for integrated care and joined-up services for older people [44], and programmes have been targeted at referral of medical care patients to social services (e.g., [45]). Potential savings in medical care services that follow such referrals might compensate for increased costs to the social care budget. There is therefore a need for more emphasis on the issue of unmet need in older people within the education of medical and nursing practitioners and their own significant role in appropriate onward referral.

### 4.3. Conclusions

This study provides valuable insights into the personal, interpersonal, and social factors related to informal care-receipt in older people, a topic that has received relatively little attention. The experience of care-receipt can last for a substantial amount of an older person's life and its nature and content impact significantly on quality of life. The present study indicates that the need for and receipt of informal care in an older person is connected to dimensions of, as well as risk factors for, social exclusion. In our model, as older people's scores on dimensions of and risk factors for social exclusion increased so did the odds of them being care-receivers, or being nonreceivers with need. Very little evidence emerged that the receipt of care or assurance was associated with social inclusion. Our findings suggest that access to informal care is important to consider when studying social exclusion processes, in that the receipt of informal care may be a marker of social exclusion, that is, that older people relying on informal care may not be integrated into society and may have unmet social needs.

## Figures and Tables

**Table 1:** Care-receipt status and sociodemographic characteristics of the sample (*N* = 1255).

Variable	
Care-receipt status: *n* (%)	
Care-receiver	269 (21.8)
Assurance-receiver	297 (24.1)
Nonreceiver with need	68 (5.5)
Nonreceiver without need	599 (48.6)
Age: M (SD), range	75.7 (7.29), 65–101
Gender: *n* (%)	
Women	776 (61.8)
Men	479 (38.2)
Marital status: *n* (%)	
Married, cohabiting	557 (44.6)
Single, divorced, separated, and never married	148 (11.8)
Widowed	545 (43.6)
Education level: *n* (%)	
Low	931 (74.2)
Medium to high	324 (25.8)
Area of residence: *n* (%)	
Urban area	627 (50.0)
Rural area	628 (50.0)

*Note*. Due to missing data *n* = 1250 for age; *n* = 1253 for coresident status.

**Table 2:** Associations between care-receipt and social exclusion dimensions, risk factors of social exclusion, and formal care receipt (*N* = 1233).

	Care-receivers (*n* = 269)	Assurance-receivers (*n* = 297)	Nonreceivers with need (*n* = 68)	Nonreceivers without need (*n* = 599)	Test statistics, significance
*Social Exclusion Variables*					
Income comfort: M (SD) (high score = low comfort)	2.72 (0.80)	2.63 (0.87)	2.93 (1.00)	2.53 (0.81)	*F*(3,1218) = 6.79, *p* < .001
Family contact: M (SD)	1.87 (1.08)	1.86 (1.18)	1.13 (1.13)	1.67 (1.17)	*F*(3,1189) = 9.01, *p* < .001
Nonfamily contact: *n* (%)					
None	78 (29.3)	93 (31.4)	27 (39.7)	180 (30.3)	*χ* ^2^(6) = 9.34, *p* = .156
Friends or neighbours	105 (39.5)	107 (36.1)	30 (44.1)	247 (41.5)	
Friends and neighbours	83 (31.2)	96 (32.4)	11 (16.2)	168 (28.2)	
Caregiving: *n* (%)	34 (12.7)	30 (10.1)	5 (7.4)	81 (13.6)	*χ* ^2^(3) = 3.82, *p* = .282
Social Loneliness: M (SD)	1.69 (1.58)	1.38 (1.58)	2.79 (2.04)	1.26 (1.50)	*F*(3,1217) = 21.2, *p* < .001
Emotional Loneliness: M (SD)	1.95 (1.91)	1.86 (2.00)	2.85 (2.29)	1.23 (1.68)	*F*(3,1206) = 22.8, *p* < .001
Perceived Community Trust: M (SD)	10.9 (2.17)	11.7 (1.59)	10.4 (2.36)	11.6 (1.71)	*F*(3,1217) = 18.6, *p* < .001
Perceived Community Integration: M (SD)	15.4 (2.56)	15.8 (2.20)	14.3 (3.18)	15.8 (2.22)	*F*(3,1218) = 9.49, *p* < .001
Perceived Community Security: M (SD)	8.08 (3.79)	9.79 (2.75)	7.88 (3.69)	10.2 (2.94)	*F*(3,1215) = 34.5, *p* < .001
Social, Cultural and Leisure Engagement: M (SD)	18.0 (16.7)	25.7 (17.0)	15.9 (14.5)	31.6 (18.3)	*F*(3,1128) = 43.9, *p* < .001
Sport and Outdoor Engagement: M (SD)	5.19 (8.35)	11.4 (12.2)	7.36 (9.00)	16.9 (13.8)	*F*(3,1161) = 60.5, *p* < .001

*Risk Factors for Social Exclusion*					
Age: M (SD)	77.6 (7.98)	76.9 (7.32)	77.4 (7.67)	74.1 (6.47)	*F*(3,1225) = 21.2, *p* < .001
Female gender: *n* (%)	184 (68.4)	206 (69.4)	41 (60.3)	334 (55.8)	*χ* ^2^(3) = 21.5, *p* < .001
Living alone: *n* (%)	138 (51.3)	206 (69.6)	53 (77.9)	231 (38.6)	*χ* ^2^(3) = 97.3, *p* < .001
Low education level: *n* (%)	238 (88.5)	224 (75.4)	54 (79.4)	399 (66.6)	*χ* ^2^(3) = 47.9, *p* < .001
Living in urban area: *n* (%)	174 (64.7)	149 (50.2)	47 (69.1)	247 (41.2)	*χ* ^2^(3) = 51.6, *p* < .001
Duration of local residence: M (SD)	63.0 (21.7)	55.3 (26.4)	65.9 (17.3)	55.5 (22.3)	*F*(3,1222) = 10.5, *p* < .001
Self-reported health: M (SD) (high score = poor health)	3.74 (0.83)	3.16 (0.84)	3.45 (0.84)	2.83 (0.83)	*F*(3,1212) = 75.7, *p* < .001
Well-being: M (SD) (high score = high well-being)	12.0 (6.55)	13.5 (6.39)	12.7 (6.77)	16.0 (5.86)	*F*(3,1219) = 29.5, *p* < .001
Medical care-receipt: *n* (%)	202 (75.1)	185 (62.3)	53 (77.9)	312 (52.1)	*χ* ^2^(3) = 50.9, *p* < .001
Formal care-receipt:					
Personal care-receipt: *n* (%)	54 (20.2)	31 (10.5)	6 (8.82)	34 (5.68)	*χ* ^2^(3) = 42.9, *p* < .001
Practical support-receipt: *n* (%)	44 (16.4)	28 (9.5)	3 (4.41)	4 (0.66)	*χ* ^2^(3) = 82.5, *p* < .001

*Note*. Due to missing data on individual variables, *n* will vary across bivariate analyses.

**Table 3:** Summary of multinomial logistic regression analysis of variables predicting membership of category of care-receipt^a^ (*n* = 980).

Category: Variable:	Care-receivers Odds ratio (95% CI)	Assurance-receivers Odds ratio (95% CI)	Nonreceivers with need Odds ratio (95% CI)
Income comfort (low comfort)	0.78 (0.61, 1.01)	0.89 (0.71, 1.11)	1.10 (0.73, 1.64)
Family contact	1.19 (0.99, 1.43)	1.21 (1.03, 1.42)^*∗*^	0.82 (0.61, 1.10)
Social Loneliness	1.08 (0.94, 1.25)	0.97 (0.85, 1.11)	1.39 (1.12, 1.72)^*∗*^
Emotional Loneliness	0.99 (0.87, 1.11)	1.16 (1.04, 1.29)^*∗*^	1.18 (0.99, 1.41)
Perceived Community Trust	0.87 (0.77, 0.99)^*∗*^	1.04 (0.91, 1.18)	0.86 (0.72, 1.03)
Perceived Community Integration	1.09 (0.99, 1.21)	1.04 (0.95, 1.14)	0.97 (0.85, 1.12)
Social, Cultural and Leisure Engagement	0.98 (0.97, 1.00)^*∗*^	1.00 (0.99, 1.01)	0.98 (0.96, 1.00)
Sport and Outdoor Engagement (0)	4.79 (2.46, 9.34)^*∗*^	1.85 (1.06, 3.24)^*∗*^	1.10 (0.37, 3.25)
Sport and Outdoor Engagement (1)	1.93 (1.05, 3.56)^*∗*^	1.71 (1.10, 2.67)^*∗*^	1.06 (0.42, 2.70)
Age	1.06 (1.03, 1.09)^*∗*^	1.05 (1.03, 1.08)^*∗*^	1.07 (1.02, 1.12)^*∗*^
Gender (female)	0.77 (0.51, 1.16)	0.63 (0.43, 0.90)^*∗*^	0.68 (0.35, 1.29)
Education Level	1.54 (0.86, 2.74)	0.98 (0.63, 1.51)	0.99 (0.42, 2.35)
Area of residence (rural)	0.38 (0.24, 0.59)^*∗*^	0.49 (0.31, 0.67)^*∗*^	0.26 (0.12, 0.56)^*∗*^
Duration of local residence	1.00 (0.99, 1.01)	0.99 (0.98, 0.99)^*∗*^	1.01 (0.99, 1.02)
Self-reported health (poor)	2.54 (1.88, 3.43)^*∗*^	1.30 (1.01, 1.67)^*∗*^	1.10 (0.73, 1.64)
Well-being	0.99 (0.95, 1.03)	0.97 (0.94, 1.01)	1.00 (0.94, 1.06)
Medical care-receipt	2.81 (1.85, 4.28)^*∗*^	1.51 (1.06, 2.15)^*∗*^	5.82 (2.68, 12.67)^*∗*^

^a^Reference category = nonreceivers without need. ^*∗*^ *p* < .05.
